# Comparison of five-year survival rates among patients with oral squamous cell carcinoma with and without association with syphilis: a retrospective case-control study

**DOI:** 10.1186/s12885-022-09583-4

**Published:** 2022-04-26

**Authors:** Moritz Hertel, Leonie Hagedorn, Andrea Maria Schmidt-Westhausen, Henrik Dommisch, Max Heiland, Robert Preissner, Saskia Preissner

**Affiliations:** 1 Department of Oral and Maxillofacial Surgery, Charité - Universitätsmedizin Berlin, Corporate member of Freie Universität Berlin, Humboldt-Universität zu Berlin, and Berlin Institute of Health, Augustenburger Platz 1, 13353 Berlin, Germany; 2 Department of Periodontology, Oral Medicine and Oral Surgery, Charité - Universitätsmedizin Berlin, Corporate member of Freie Universität Berlin, Humboldt-Universität zu Berlin, and Berlin Institute of Health, Aßmannshauser Str. 4-6, 14197 Berlin, Germany; 3 Institute of Physiology and Science-IT, Charité - Universitätsmedizin Berlin, Corporate member of Freie Universität Berlin, Humboldt-Universität zu Berlin, and Berlin Institute of Health, Philippstr. 12, 10115 Berlin, Germany

**Keywords:** Oral malignant neoplasia, Oral squamous cell carcinoma, Syphilis, Premalignant condition, Mortality, Survival rate, Multi-center data

## Abstract

**Background:**

Syphilis is an infectious disease that is at least discussed to be premalignant. This potential, combined with its general pathological impact, raises the question if syphilis increases mortality in oral cancer patients. The aim of the study was to assess if the five-year survival rates among patients suffering from oral squamous cell carcinoma (OSCC) with (cohort I) and without association with syphilis (cohort II) differ.

**Methods:**

Retrospective clinical data of patients diagnosed with OSCC (International Classification of Diseases [ICD]-10 codes C01–06) within the past 20 years from the access date September 25, 2021 were retrieved from the TriNetX network (TriNetX, Cambridge, Massachusetts, USA) to gain initial cohort 0. Subjects also diagnosed with syphilis (ICD-10 codes A51–53) were assigned to cohort I. Cohort II was comprised of the remaining individuals of cohort 0 by creating a group with the same number of patients as cohort I, and by matching for age and gender. Subsequently, Kaplan-Meier analysis and Cox proportional hazards regression were performed, and risk, odds and hazard ratios were calculated.

**Results:**

Of a total of 73,736 patients in cohort 0, 199 individuals were each assigned to cohort I and II. During the five-year period after tumor diagnosis, 39 and 30 patients in cohort I and II died. The five-year survival probabilities did not significantly differ between the cohorts (I vs. II = 74.19% vs. 75.01%; *p* = 0.52; Log-Rank test), nor the risk of dying (I vs. II = 19.6% vs. 15.08%; risk difference = 4.52%; *p* = 0.23). The calculated risk, odds and hazard ratios were 1.3 (95% confidence interval [CI] = 0.84; 2.00), 1.37 (95% CI = 0.81; 2.31) and 1.17 (95% CI = 0.73; 1.88), respectively.

**Conclusions:**

The obtained results indicate that the survival rate of individuals with OSCC might not be negatively influenced if syphilis is present/associated. However, the results need to be interpreted cautiously due to limitations related to the retrospective approach, especially as data on the tumor staging were not accessible.

**Trial registration:**

Due to the retrospective nature of the study, no registration was necessary.

## Background

Syphilis is an infectious disease caused by the bacteria *Treponema pallidum, subsp. pallidum*. It is mostly transmitted sexually [1]. All stages of the disease can cause maxillofacial, especially oral, manifestations [2]. Even though the authors of the recent World Health Organization (WHO) classification did not find sufficient evidence to classify syphilis as an oral potentially malignant disorder (OPMD) [3], it was at least controversially discussed whether oral lesions caused by syphilis, as well as the disease itself, could be associated with an augmented risk of developing different malignant neoplasia, inter alia oral squamous cell carcinoma (OSCC) [4–9]. Among the key alterations fundamental to cancer cell development [10], syphilis might especially establish a tumor-promoting inflammation and trigger sustaining proliferative signaling. Besides the fact that the presence of syphilis might enhance the risk of developing tumors, it also acts as a co-morbidity, regardless of its questionable malignant character. Both features might potentially impede outcomes in patients with OSCC. Accordingly, the purpose of the present study was to analyze if the five-year survival rates among patients with oral cancer with and without association with syphilis differed. In the recent literature, this question has not yet been addressed. It was hypothesized that the survival rate of subjects suffering from OSCC associated with syphilis was significantly higher compared to individuals with oral cancer without syphilis.

Regardless of its rising incidence within the last decades in specific cohorts in high-income countries, syphilis is still a relatively rare disease [1]. The TriNetX Global Health Research Network (TriNetX, Cambridge, Massachusetts, USA) was chosen to retrieve related retrospective data, as it provides access to a significant number of medical records. TriNetX is a database that includes clinical data from more than 120 health care organizations (HCOs) from 19 countries. Its purpose is to enable HCOs, contract research institutes and biopharmaceutical companies to access and exchange longitudinal clinical data, and to provide state-of-the-art analytics. By September 2021, TriNetX had collected electronic medical records from more than 250 million patients. It has previously been used to research medical topics of worldwide interest, including the coronavirus disease 2019 (COVID-19) pandemic [11, 12].

## Patients and methods

### Data acquisition, inclusion and exclusion criteria

**Fig. 1:**
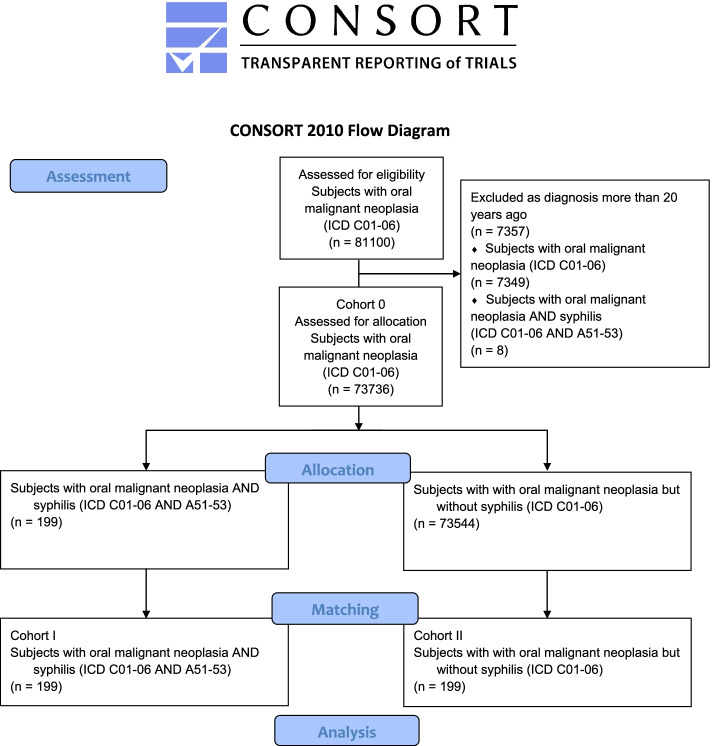
Modified CONSORT flow chart

The TriNetX network was accessed on Saturday, September 25, 2021; whereby the eligibility period was limited to the previous 20 years from the access date to take account of recent developments in the diagnosis and therapy of OSCC. The database was searched for patients who were diagnosed with OSCC (International Classification of Diseases [ICD]-10 codes C01–06) between five and 20 years before the access date. Subjects with malignant odontogenic tumors or neoplasia of the lips, salivary glands, tonsillae, oro-, naso and hypopharynx, recessus piriformis, as well as tumors located in other areas different from the oral cavity (ICD-10 codes C00, 07–14, 41.02–41.1, 43 and 44) were not included. The obtained cohort 0 was then tested for diagnosis with early, late or not further specified syphilis (ICD-10 codes A51–53). Patients with syphilis connata (ICD-10 code A50) were not included in the analysis. In order to mitigate confounder bias via propensity score, stratified and balanced sub-cohorts across current age, age at tumor diagnosis and gender distribution were retrieved from the initial cohorts. One-to-one matching was applied in order to replicate randomized conditions as closely as possible by obtaining cohorts with similar covariate distributions. All individuals diagnosed with both ICD-10 codes C01–06 and A51–53 were assigned to cohort I (patients suffering from OSCC and syphilis). Cohort II (subjects with OSCC, but without syphilis) was subsequently obtained from the remaining individuals within cohort 0, and by matching as shown in the CONSORT flow chart (Fig. 1). The final cohorts were furthermore checked for human papillomavirus (HPV) and human immunodeficiency virus (HIV) diagnoses. HPV was diagnosed by means of testing for HPV-DNA in tumor samples, whereas HIV diagnoses were based on serological testing (enzyme-linked immunosorbent assay [ELISA] and HIV antibody test).

### Data analysis

After defining the primary outcome as “death”, Kaplan-Meier survival analysis and Cox proportional hazards regression were performed, and risk ratios (RR), odds ratios (OR), as well as hazard ratios (HR), were calculated for the respective cohorts. The evaluation was limited to a period of 5 years after cancer diagnosis as patients are considered to be healed in the case of absence of OSCC and metastases after the respective time. Statistical analysis was performed applying the Log-Rank test, whereby *p* ≤ 0.05 was defined as a significance threshold.

## Results

### Assessment, allocation and matching

A total eligible number of 81,100 patients from 65 health care organizations from eleven countries with OSCC (ICD-10 codes C01–06) were retrieved from the database. The number of subjects who were excluded due to diagnosis with either ICD-10 codes C01–06 or A51–53 over the 20 years before the access date were eight (for OSCC and syphilis) and 7349 (for OSCC, but no syphilis). As a result, a total of 73,736 individuals with OSCC were available for allocation to cohort 0. Of those, 199 patients were also diagnosed with syphilis (ICD-10 codes A51–53) and assigned to cohort I (females: 44 [22.11%]; males: 155 [77.89%]; mean(±standard deviation) current age(±standard deviation) = 62.79 ± 13.52 years; mean age at diagnosis = 57.51 ± 13.64 years). The same number of subjects were assigned by matching of both groups to cohort II, as explained above (females: 43 [21.61%]; males: 156 [78.39%]; mean current age = 62.90 ± 13.31 years; mean age at diagnosis = 57.61 ± 13.43 years). The groups did not differ significantly in gender distribution or age (*p* = 0.90 and 0.93; Log-Rank test). The obtained propensity score was 0.98. Table 1 shows the patient characteristics of both cohorts before and after matching.

**Table 1:** Patient characteristics before and after matching of cohorts I (ICD-10 codes C01–06 and A51–53) and II (ICD-10 codes C01–06 without A51–53)

Patients (n)	Before matching				After matching			
	Cohort I	Cohort II	***p-*** value	Standardized mean difference	Cohort I	Cohort II	***p-***value	Standardized mean difference
Total	199	73,544			199	199		
Male	155 (77.89%)	49,015 (66.65%)	< 0.05	0.2531	155 (77.89%)	156 (78.39%)	0.90	0.0121
Female	44 (22.11%)	24,488 (33.25%)	< 0.05	0.2519	44 (22.11%)	43 (21.61%)	0.90	0.0121
**Mean current age (years)**	62.79	68.18	< 0.05	0.3956	62.79	62.90	0.93	0.0082
Standard deviation	13.52	13.73			13.52	13.31		
Minimum	7	0			7	7		
Maximum	90	90			90	90		
**Mean age at diagnosis**	57.51	61.94	< 0.05	0.3207	57.51	57.61	0.93	0.0077
Standard deviation	13.64	13.97			13.64	13.43		
Minimum	4	0			4	4		
Maximum	90	90			90	90		

Percentage refers to gender distribution within the respective cohorts. *P*-value refers to comparison between both cohorts (Log-Rank test)

*Abbreviations*: *ICD* International Classification of Diseases

### Patient survival

**Fig. 2:**
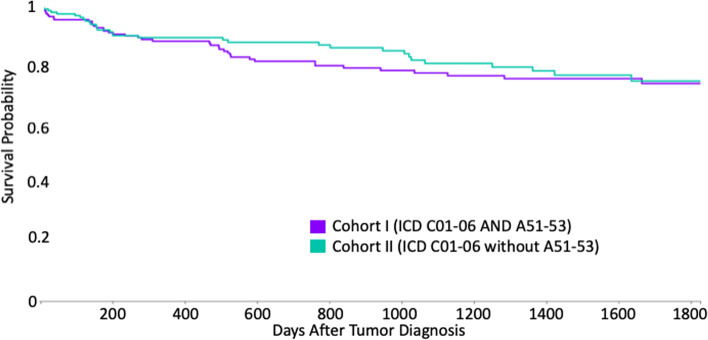
Kaplan-Meier survival curve of cohorts I (ICD-10 codes C01–06 and A51–53) and II (ICD-10 codes C01–06 without A51–53)

**Fig. 3:**
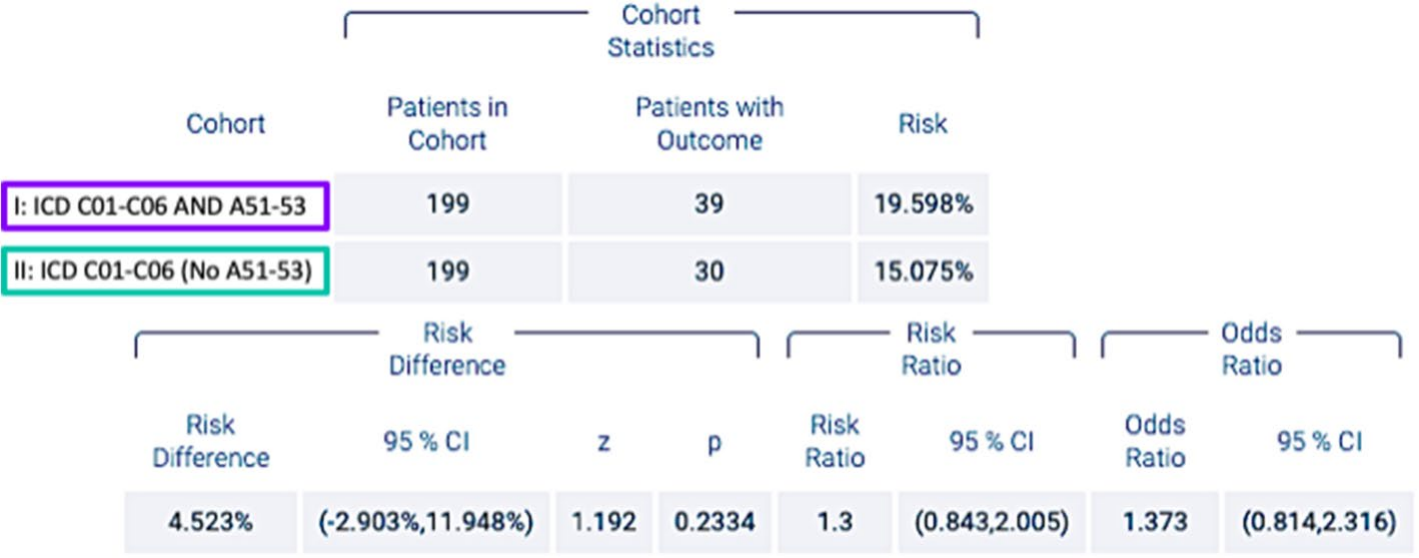
Risk of death, risk ratios and odds ratios of cohorts I (ICD-10 codes C01–06 and A51–53) and II (ICD-10 codes C01–06 without A51–53)

During the five-year period after diagnosis of OSCC, 39 patients in cohort I died, whereas 30 individuals in cohort II passed away, which corresponds to risks of death of 19.59 and 15.07%, respectively. The difference between the cohorts was not statistically significant (*p* = 0.233; Log-Rank test). Accordingly, the survival probability at the end of the time window was 74.19 and 75.01%, respectively, for subjects among cohorts I and II (Fig. 2). In addition, no statistical significance was found (*p* = 0.52). The related RR, OR and HR were 1.3 (95% confidence interval (CI) lower: 0.84 and upper: 2.00), 1.37 (95% CI lower: 0.81 and upper: 2.31) and 1.17 (95% CI = 0.73; 1.88), respectively, as shown in Fig. 3.

### HPV and HIV infection

Both cohorts were negatively tested for difference in distribution to HPV diagnoses (*p* > 0.05). However, significantly more cases of HIV infection were present in cohort I (*n* = 56; females: 10 and males: 46) compared to cohort II (*p* <  0.05). Therefore, subcohorts that were matched for the presence of HIV, as well as for gender and age distribution within the infected, and which consisted of 55 patients each, underwent statistical testing again. In accordance with the results presented above, the risks of death and survival probabilities did not differ significantly between those subcohorts (*p* > 0.05).

## Discussion

The aim of the present work was to evaluate if the five-year survival rates among patients suffering from OSCC and syphilis differed from individuals with oral cancer, but without syphilis. This study was the first to address this question by retrospectively evaluating data from multiple centers to investigate larger cohorts. Different from the hypothesis expressed in the introduction, it was found that the survival rates of individuals with OSCC did not differ significantly if syphilis was parallelly diagnosed, compared to subjects without syphilis. At least, OR was found to be 1.3, which indicates a trend in accordance with the hypothesis. Furthermore, the survival curves of the investigated cohorts show intermediary discrepancies before they align at the end of the time window (Fig. 2).

The obtained results certainly support the recent WHO classification, according to which syphilis in no longer seen as an OPMD due to the lack of sufficient evidence of any premalignant character [3]. Even authors who discussed the role of the disease in oral cancer, and attributed syphilis with an etiologic factor in tumorigenesis, described it as one of relatively moderate impact [5, 6]. Despite the fact that syphilis itself acts as a co-morbidity, especially in its early stages, it can be sufficiently and cost-effectively treated with antibiotics [13]. The availability of a prompt curative treatment, at least in mid- and high-income countries, might limit the pathological potential of syphilis in this regard. However, the retrieved results need to be discussed regarding limitations that stem from the specific design of the present study. Specifically, the TriNetX database can be searched for patients with specific diagnoses by using the respective ICD-10 codes, which implies that oral malignant neoplasia are classified according to their localization. The vast majority of these tumors were supposedly OSCCs, as the most frequent malignant tumor of the oral cavity [5, 14, 15]. In addition, certain malignant entities of odontogenic, lymphatic or osseous origin, for example, are classified separately within the ICD-10, and have therefore been excluded. Nevertheless, patients suffering from different subtypes of OSCCs or even other rare oral malignant neoplasia (e.g., oral malignant melanoma) might have been included in the investigated cohorts. This could have influenced the obtained results as different malignant (sub-)entities show varying biological and pathological features, such as proliferation rate or specific risk of developing metastases [16]. Furthermore, different characteristics have been identified to influence the survival rates of patients with oral malignant neoplasia, even though other features are still discussed controversially [10]. Localization and the staging of the tumor disease according to the Union Internationale Contre le Cancer (UICC), including clinical, histological/pathological and molecular features, as well as the applied therapies, have especially been found to significantly influence patient probability of survival [5, 17–23]. Smoking behavior, alcohol abuse and the presence of human papilloma virus are not only etiological factors, but also factors that impact the prognosis of patients with oral cancer [23, 24]. Ideally, the investigated cohorts would have been matched for UICC stage. From the TriNetX network, no respective data was available. However, features related to the lacking information might have influenced the mortality of patients with OSCC/syphilis within the investigated cohorts. In contrast, matching of the compared cohorts for age and gender might have levelled out the differences in distribution of these variables, at least to a certain extent. Due to the limited availability of certain data, the percentage of patients of cohort II who tested negatively for syphilis also could not be assessed. Supposedly, testing was not carried out routinely, at least in cases without respective suspicion. Therefore, uncertainty remains that undetected cases of syphilis might have been included in cohort II. Despite the lack of the mentioned information, the quality of the data retrieved from the TriNetX database can be classified as high. The database even matches the strict conditions of the National COVID Cohort Collaborative N3C, which was formed to accelerate the understanding of SARS-CoV-2, and of which TriNetX recently became a part.

To overcome the aforementioned limitations, future research might consider using a prospective approach applying standardized therapies, including routine testing for syphilis, to evaluate if the presented results can be confirmed thus far. In addition, histological/pathological and molecular data may be included into the analysis. Despite this, the relatively low incidence of syphilis remains problematic regarding an appropriate prospective study design [1].

## Conclusions

Syphilis is no longer classified as OPMD within the recent WHO classification [3], even though its premalignant potential has at least been discussed. Despite its questionable role as a contributor to oral cancer, and its impact as a co-morbidity, the present study did not show a negative influence of syphilis on the five-year survival rate of patients with OSCC, compared to patients without syphilis. However, these results should be cautiously interpreted due to limitations related to the applied approach.

## Data Availability

The datasets used and analyzed can be retrieved from the TriNetX network (https://trinetx.com). Public access to the database is closed. If no access is available, the datasets can be retrieved from the corresponding author based on reasonable request.

## Abbreviations

ELISA: Enzyme-linked immunosorbent assay
HCO: Heath care organization
HIV: Human immunodeficiency virus
HPV: Human papillomavirus
HR: Hazard ratio
ICD: International Classification of Diseases
OR: Odds ratio
OPMD: Oral potentially malignant disorder
OSCC: Oral squamous cell carcinoma
RR: Risk ratio
UICC: Union Internationale Contre le Cancer
WHO: World Health Organization
